# Using street view imagery to examine the association between urban neighborhood disorder and the long-term recurrence risk of patients discharged with acute myocardial infarction in central Beijing, China

**DOI:** 10.1016/j.cities.2023.104366

**Published:** 2023-05-17

**Authors:** Yuyang Zhang, Qiuju Deng, Moning Guo, Yan Li, Feng Lu, Jingjia Chen, Jiayi Sun, Jie Chang, Piaopiao Hu, Ningrui Liu, Jing Liu, Ying Long

**Affiliations:** a School of Architecture, Tsinghua University, Beijing 100084, China; b Department of Epidemiology, Beijing An Zhen Hospital, Capital Medical University, Beijing Institute of Heart, Lung and Blood Vessel Diseases, the Key Laboratory of Remodeling-Related Cardiovascular Diseases, Ministry of Education, and the Beijing Municipal Key Laboratory of Clinical Epidemiology, Beijing 100029, China; c School of Architecture and Hang Lung Center for Real Estate, Key Laboratory of Eco Planning & Green Building, Ministry of Education, Tsinghua University, 100084, China; d Beijing Municipal Health Commission Information Center (Beijing Municipal Health Commission Policy Research Center), Beijing, China

**Keywords:** Recurrent acute myocardial infarction (AMI), Built environment, Cardiovascular disease, Prognosis, Risk factor

## Abstract

**Background:**

To examine the association between urban neighborhood disorder and the recurrence risk of patients with acute myocardial infarction (AMI) in central Beijing, China.

**Methods:**

Recurrent AMI was identified by the Beijing Monitoring System for Cardiovascular Diseases through the end of 2019 for patients discharged with AMI between 2007 and 2017. Cox proportional hazards models were performed to estimate associations between neighborhood disorder and AMI recurrence.

**Results:**

Of 66,238 AMI patients, 11,872 had a recurrent event, and 3117 died from AMI during a median followup of 5.92 years. After covariate adjustment, AMI patients living in the high tertile of neighborhood disorder had a higher recurrence risk (hazard ratio [HR] 1.08, 95 % confidence interval [CI], 1.03–1.14) compared with those in the low tertile. A stronger association was noted for fatal recurrent AMI (HR 1.21, 95 % CI 1.10–1.34). The association was mainly observed in females (HR 1.04, 95 % CI: 1.02 to 1.06).

**Conclusions:**

Serious neighborhood disorder may contribute to higher recurrence risk, particularly fatal recurrence, among AMI patients. Policies to eliminate neighborhood disorders may play an important role in the secondary prevention of cardiovascular disease.

## 1 Introduction

Exposure to hazardous neighborhood built environments can result in higher levels of environmental exposure (such as air quality and noise), emotional stress, sedentary behavior, obesity, and high blood pressure, and subsequently influence cardiovascular health (Malambo et al., 2016; Zhang et al., 2023). However, Previous studies regarding built environments have mainly focused on the quantitative use of distance, density and diversity to estimate the exposure level in terms of quantity. Few studies have assessed the quality of the built environment, which is more ubiquitous and visually obvious (Dempsey, 2008). Its negative features (e.g., abandoned buildings, cracks on roads, and vacant lots covered by weeds and trash) represent the decline of the spatial quality in the spatial dimension, which partly coincide with the features used to evaluate neighborhood disorder (Marco et al., 2015; Ndjila et al., 2019; Ross & Mirowsky, 1999). Previous studies regarding built environments have mainly focused on the quantitative use of distance, density and diversity to estimate the exposure level in terms of quantity. A few studies have assessed the quality of the built environment, such as neighborhood disorder, which is more ubiquitous and visually obvious (Dempsey, 2008). However, the “neighborhood disorder” (Parajara et al., 2020), which has traditionally been used in social studies mainly describe issues related to violence, crime, and safety (Barber et al., 2016; Robinette et al., 2018; Robinette et al., 2018; Tamura et al., 2019; Jansåker et al., 2022). However, we utilized the concept of “neighborhood disorder” and modified its characteristics to better align with our focus on the quality of the built environment in our study area. This disorder phenomenon reflects the prevalence of informal and undermaintained urban landscapes (Ross & Mirowsky, 1999). In China, it may be caused by extensive urban spatial expansion as well as the lack of corresponding regulations and management measures (Chen & Long, 2021), which is distinctive from Western countries that are caused by racial separation and low individual socioeconomic status (Plascak et al., 2021).

With the increase in the survival rates after acute myocardial infarction (AMI) (Plakht et al., 2016; Yamamoto et al., 2019), an increasing number of patients are being discharged from the hospital and returning to neighborhood life, inevitably being exposed to the built environment in their neighborhood area. Previous studies have identified pathways between the neighborhood built environment and major cardiovascular diseases in terms of distance, density and diversity, including physical activity, stress, air quality and noise (Malambo et al., 2016; Zhang et al., 2023). It is therefore important to understand the impact of the quality of the built environment on the secondary prevention of cardiovascular disease. A recent meta-analysis showed that neighborhood disorder was associated with mental health, substance use, and overall health status (O’Brien et al., 2019a). However, few studies have examined the effect of neighborhood disorder on patients discharged with AMI.

The objective of this study was to examine whether neighborhood disorder was associated with the recurrent risk of patients discharged with AMI and whether the associations varied by characteristics of patients and residential areas in a city-wide patient cohort based on the Beijing Monitoring System for Cardiovascular Diseases.

## 2 Methods

### 2.1 Data source and study population

This was a patient cohort study based on data obtained from the Beijing Monitoring System for Cardiovascular Diseases, which links routinely collected records from the Beijing Hospital Discharge Information System with the Beijing Vital Registration Monitoring System (Xie et al., 2015) through the personal identity number that is unique for all Chinese citizens. After linking, personal identity numbers were replaced by unique study numbers, and all variables with identification were masked in all data files.

**Fig. 1:**
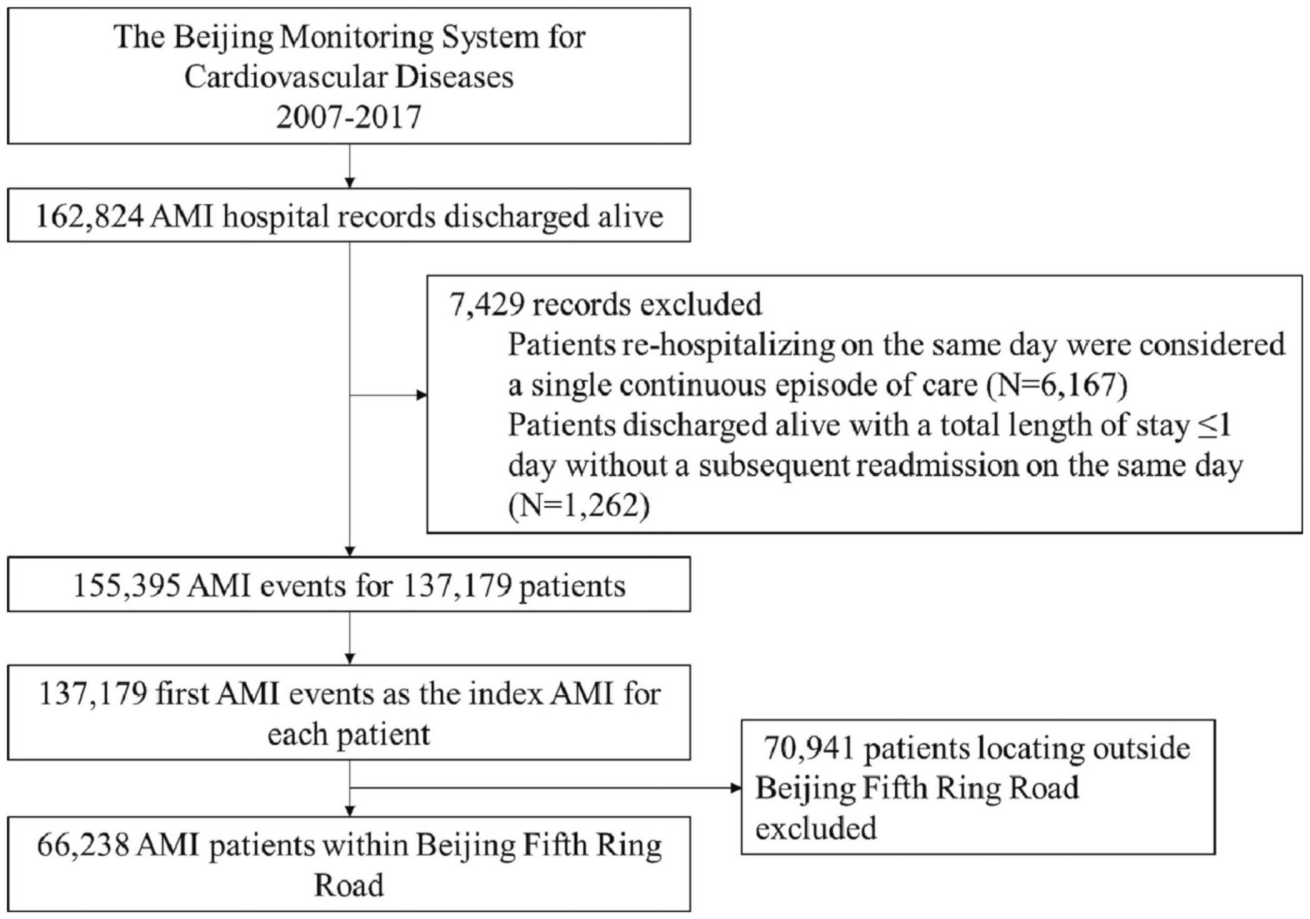
Flowchart of the study population. AMI = acute myocardial infarction.

Participants in this study referred to patients discharged alive with a primary diagnosis of AMI (the International Classification of Diseases Tenth Revision [ICD-10] codes of I21 or I22) between January 1, 2007 and December 31, 2017 who were permanent residents in Beijing. A total of 162,824 hospital discharge records with AMI were identified. Patients who were discharged and then rehospitalized on the same day were considered to have a single continuous episode of care (N = 6167). We also excluded patients who were discharged alive with a total length of stay ≤1 day without a subsequent readmission on the same day (n = 1262) because they were unlikely to have had an AMI. If a patient had >1 AMI event during the study period, we selected the first event during the study period as the index AMI (18,216 events excluded). Due to the availability of data on neighborhood disorders, a total of 66,238 AMI patients located within Beijing Fifth Ring Road (known as central Beijing area) were included in the final analysis (Fig. 1).

This study was performed in accordance with the principles of the Declaration of Helsinki and approved by the Ethics Committee of Beijing An Zhen Hospital (ks2020010) with a waiver of informed consent.

### 2.2 Measurement of neighborhood disorder

We first geocoded the address of patients into spatial coordinates and imported them into geographic information systems (GIS) software ArcMap (ESRI, Redlands, CA). Then, we generated a 500-meter Euclidian buffer centered at each patient’s residence to measure neighborhood disorder and contextual covariates. The 500 m buffer considered mobility, i.e. being within walking distance (<10 min), which is an area of regular activity for neighborhood residents (Villeneuve et al., 2012). Meanwhile, among different types of buffers (i.e., Euclidian, Network, Sausage, Convex hull), only the Euclidean distance buffer can measure the walking range to the greatest extent and fully encompass the actual walking range under the same spatial scale (Duncan et al., 2021). Therefore, 500-meter Euclidian buffers were finally selected.

Referring to previous studies (Sampson & Raudenbush, 1999; Quinn et al., 2016), we inclusively listed the neighborhood disorder features and built a checklist. The final list contained a total of five domains of 19 disorder features that described a broken or decay condition of spatial elements along the streets, including architecture, retail, greening, road, and other infrastructure. Then, four auditors with professional backgrounds in architecture or urban planning were recruited to finalize the checklist (Table S1) after reviewing a large number of street view images.

We divided 16,790 streets with intervals of 50 m and downloaded street view images of four directions from the Tencent Map API, namely, north, west, east, and south within the Beijing Fifth Ring Road (most of the streets in this area are E-W or N-S). In total, we captured 281,745 images from 2015 for 70,437 locations. To identify the disorder features, we built a virtual audit platform in which four directions’ street view images for each location were displayed on the same page (Fig. S1). First, 10 % of all the street view images were randomly chosen for four auditors to separately mark. The interrater reliability (Kappa) value of the pre-evaluation results was 0.83 (Chen et al., 2020). Next, the locations were randomly and equally distributed to four auditors to minimize the regional differences caused by auditors’ subjectivity. Third, the neighborhood disorder value for each location was measured as the total number of disorder features in four directions (Chen & Long, 2021). Finally, the patient’s neighborhood disorder exposure level was measured by the average neighborhood disorder value of all the locations within the 500-meter buffer. Higher neighborhood disorder values represented more serious neighborhood disorder environment exposure for patients.

### 2.3 Covariates

The study’s covariates draw from three crucial categories: the built environment, natural environment, and individual information - all essential factors in determining CVD outcomes. The built environment covariates included proximity to green space and main roads, with clear evidence of their impact on CVD outcomes (Malambo et al., 2016; Zhang et al., 2023). Spatial heterogeneity of north and south within Beijing required residential location to be included as a covariate. The natural environment was represented by the harmful effects of high PM_2_._5_ concentration exposure (Lu et al., 2015). The final multivariate models utilized a range of covariates, including age, sex, marital status, AMI type, CHD history, comorbidities, residential location, proximity to parks and main roads, and PM_2_._5_ exposure levels. Individual information mainly include available covariates that were associated with CVD outcomes, including age, sex, marital status, AMI type, CHD history, comorbiditieso. In addition, the sensitivity analysis additionally adjusted neighborhood-level household income (Ohm et al., 2021) and driving distance to the nearest percutaneous coronary intervention (PCI)-capable hospital (Su et al., 2023).

Information about age, sex, marital status, type of AMI, history of CHD, and comorbidities was acquired from the Beijing Monitoring System for Cardiovascular Diseases. Age was coded in two groups: the young and middle-aged group (≤65 years) and the older group (>65 years). Marital status was categorized as married and nonmarried. AMI events were further classified as ST-segment elevation myocardial infarction (STEMI) (codes of I21.0–I21.3, I22.0, I22.1, and I22.8), non-ST-segment elevation myocardial infarction (NSTEMI) (code of I21.4), and unspecified AMI (codes of I21.9 and I22.9). Comorbidities, including dyslipidemia, diabetes mellitus, and hypertension, were extracted from the secondary discharge diagnosis code.

The main gates of parks were derived from points of interest (POIs) on the Baidu Map (2011, 2014, 2016, and 2018), which included the coordinates and descriptions of all types of places and facilities in Beijing. Distance to the nearest park was defined as the Euclidean distance from the patient’s residence to the nearest park’s entrance. The main roads were derived from road network data from Amap (2011 and 2018), including grade information, such as main roads and secondary roads. Distance to the main road was defined as the Euclidean distance from the patient’s residence to the nearest main road. The PM_2.5_ concentration was predicted by relating satellite-based aerosol optical depth (AOD) retrievals to ground-based PM_2.5_ observations (Long et al., 2018) and aggregated at the subdistrict level. The PM_2.5_ concentration was assigned to the patients living in the corresponding subdistrict to represent the individual PM_2.5_ exposure level. Household income data were released by the Beijing Household Travel Survey (2010) (Beijing Municipal Commission of Transport & Beijing Transportation Research Center, 2012), which included household and personal information with a 1.36 % sampling rate of the total Beijing population. Household income was aggregated to the traffic analysis zone (TAZ) (Long & Thill, 2015) in average value to represent neighborhood-level income. The neighborhood-level income was assigned to the patients living in the corresponding TAZ. The income data were missing for 19.97 % of patients, as those patients living in the area were not covered by the survey. The central Beijing area was divided into two parts by Chang’an Avenue, namely, northern and southern parts. Residential area focused on the location of patients’ residences. Data from PCI-capable hospitals (2007–2012) were provided by the Beijing Municipal Health Commission Information Center. Driving distance to the PCI-capable hospital was calculated from the patient’s residence to the nearest PCI-capable hospitals based on the constructed historical road network dataset of the corresponding year. The data were only available for patients who experienced the first event in 2007–2012.

### 2.4 Follow-up and outcomes

The outcome in our study was recurrent AMI, including fatal and nonfatal events. Information on all events was systematically obtained from the Beijing Monitoring System for Cardiovascular Diseases using the index AMI discharge as time zero. Additionally, the event date was ascertained from either the initial point of diagnosis or a death certificate. Person-years of follow-up was calculated as the interval from the discharge date to the occurrence of the outcome event, the preselected date of December 31, 2019, or 5 years of follow-up.

### 2.5 Statistical analysis

Categorical variables are presented as counts and percentages, and groups were compared using χ^2^ tests. The normality of distribution was evaluated by the Kolmogorov–Smirnov test. Continuous variables are presented as the means and standard deviations (SD) or medians and interquartile range (IQR), and groups were compared using Kruskal–Wallis tests. The patients were stratified into low, middle, and high neighborhood disorders according to the tertiles in the exposure level. Demographics and other baseline characteristics were compared across these tertiles.

The cumulative incidence of recurrent AMI in the groups stratified by neighborhood disorder was calculated using the Kaplan-Meier method and compared using the log-rank test. To estimate associations between neighborhood disorder and the risk of AMI recurrence, Cox regression analyses were performed to derive unadjusted and adjusted hazard ratios (HRs) and corresponding 95 % confidence intervals (CIs). Furthermore, the effects of the two higher tertiles of exposure were estimated using the lowest exposure tertile as a reference. The initial model was unadjusted, and the second model was adjusted for patient age and sex. The third model was built on the second model by additionally adjusting the following covariates: marital status, type of AMI, history of CHD, comorbidities (dyslipidemia, diabetes mellitus, and hypertension), residential area, distance to the nearest park, distance to the main road, and PM_2.5_ exposure level. The proportional hazards assumption in each Cox model was verified by evaluating the weighted Schoenfeld residuals.

We also performed subgroup analyses to examine whether these associations differed by sex, age group (≤65 years or > 65 years), marital status (married or nonmarried), type of AMI (STEMI or NSTMI), history of CHD, dyslipidemia, diabetes mellitus, hypertension, residential location (south or north), distance to the nearest park (≤500 m or >500 m), distance to the main road (≤100 m or >100 m), and PM_2.5_ exposure level (≤88 ^μg/m3^ or >88 ^μg/m3^, according to mean exposure level). Statistical significance between subgroup-specific effects was assessed using a two-sample z-test (Altman & Bland, 2003). To assess possible effect modification, we modeled interaction terms between continuous neighborhood disorder and each potential effect modifier and used a Wald test to assess statistical significance.

Sensitivity analyses were conducted to determine the robustness of the main results. First, we additionally adjusted neighborhood-level income and driving distance to the nearest PCI-capable hospital based on the third model among patients with available data. Second, we restricted the analyses to incident AMI patients who had not been admitted because of AMI within a minimum of 5 years before the event. To ensure this, records from 2007 to 2011 were used as a wash-out period to exclude patients with a prior admission for AMI. Third, the association between neighborhood disorder and the risk of AMI recurrence at 5 years after the index event was also examined, which indicated that the follow-up time of all the included patients was 5 years, except for those patients who had the expected outcome within 5 years.

All statistical analyses were conducted with Stata version 14.2 (StataCorp) and R version 4.0.3 (R Foundation for Statistical Computing, Vienna, Austria). All tests of significance were two-tailed, with a level of significance set at an alpha of 0.05.

## 3 Results

### 3.1 Baseline patient characteristics

The mean (SD) age of the 66,238 patients discharged alive with AMI was 66.0 (13.2) years, and 29.1 % were female. Overall, patients living in the higher tertile of neighborhood disorder were younger, had a higher proportion of STEMI and comorbidities (dyslipidemia, diabetes mellitus, and hypertension), were more likely to reside in the southern part of central Beijing, had a longer distance to the nearest park and the main road, and had higher exposure levels of PM_2.5_. Additionally, the proportion of women was lower among patients living in the lowest tertile of neighborhood disorder than among those living in the two higher tertiles. No significant difference was observed in the proportion of nonmarried individuals and CHD history across neighborhood disorder tertiles (Table 1).

### 3.2 Neighborhood disorder characteristics

**Fig. 2:**
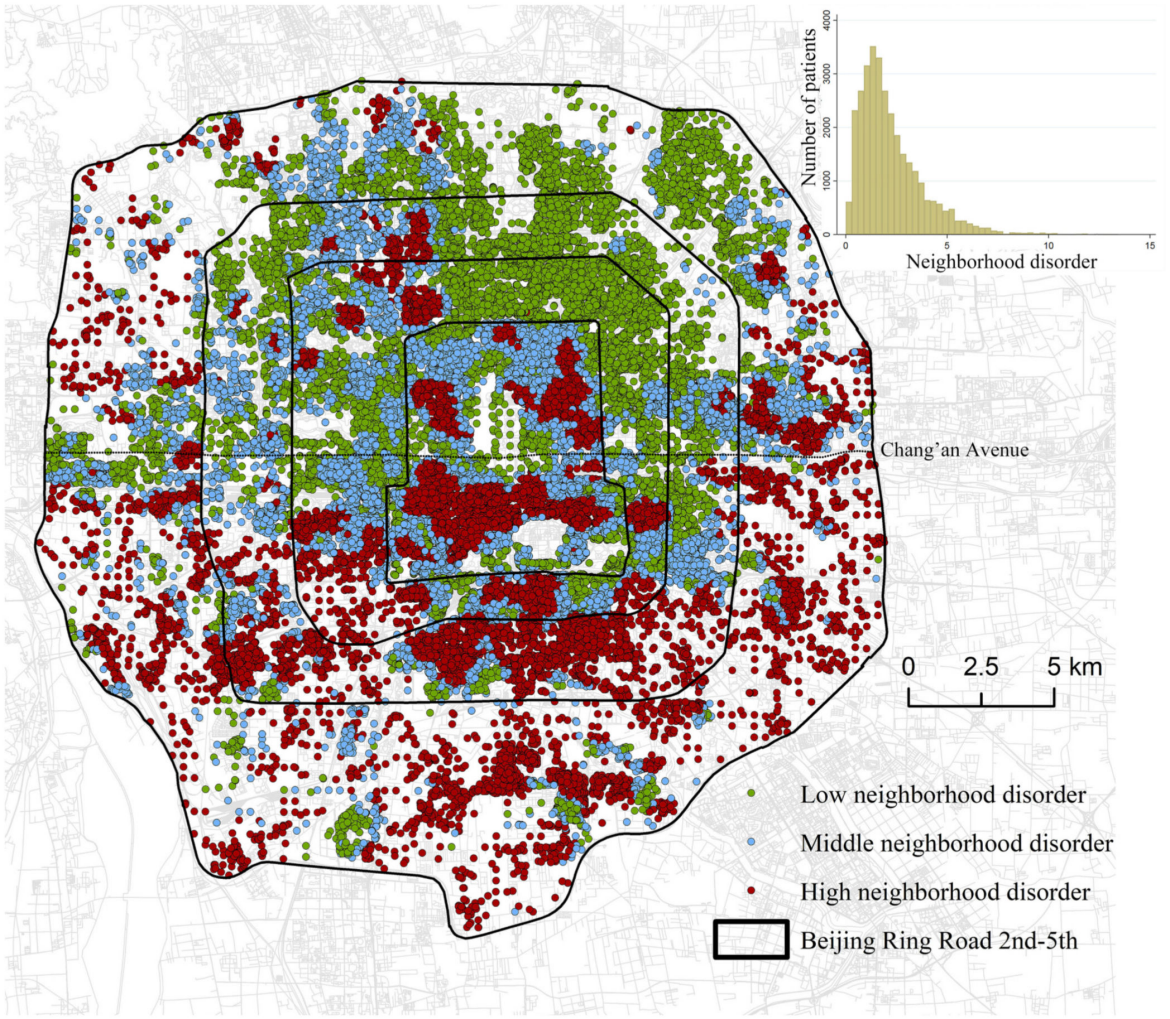
The spatial distribution of patients living in different neighborhood disorder tertiles.

The spatial distribution of AMI patients living in tertiles is presented in Fig. 2. The median neighborhood disorder in Beijing is 1.81 (IQR: 1.36–2.51). Patients living in the highest neighborhood disorder tertile were mainly distributed to the south of Chang’an Avenue. Only 23.72 % of patients living in the high neighborhood disorder tertile were distributed in the northern part of Beijing; they were mainly clustered within Beijing 2nd Ring Road. A total of 78.87 % of patients living in the lowest neighborhood disorder tertile were distributed in the northern part of central Beijing, especially clustered at the northeast corner of Beijing 2nd Ring Road.

### 3.3 Association between neighborhood disorder and recurrent AMI

In total, the median follow-up time was 5.92 years (IQR: 5.78 years) for recurrent AMI. During the follow-up, 11,872 patients had a recurrent AMI event, including 3117 fatal events and 8755 nonfatal events.

**Fig. 3:**
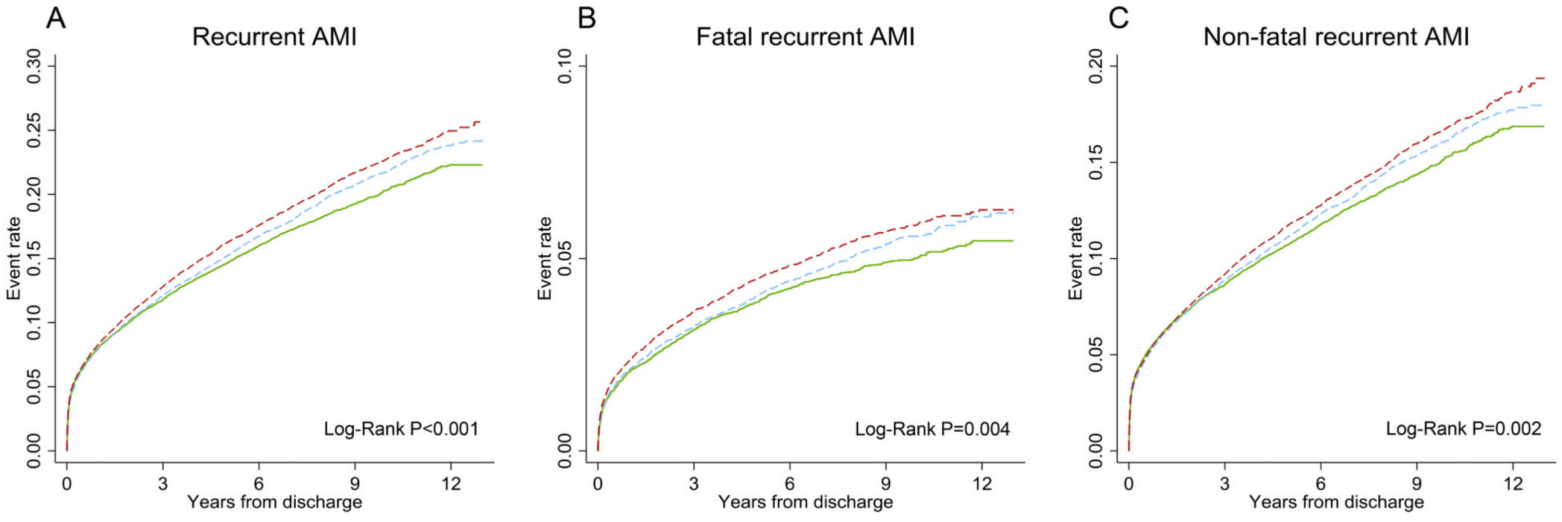
Cumulative event rates of recurrent AMI from discharge after first acute myocardial infarction. Unadjusted cumulative rate curves for (A) recurrent AMI, (B) fatal AMI, and (C) nonfatal AMI across categories of neighborhood disorder. The lowest tertile of neighborhood disorder is denoted by the solid green line. AMI = acute myocardial infarction.

Kaplan-Meier curves of the cumulative incidence of recurrent AMI from the index event among AMI patients by neighborhood disorder tertiles are shown in Fig. 3. Patients living in the higher neighborhood disorder tertile had a significantly higher cumulative incidence of recurrent AMI (*P* < 0.001), fatal recurrent AMI (*P* = 0.004), and nonfatal recurrent AMI (*P* = 0.002) after discharge for AMI throughout the entire follow-up period.

After adjusting for personal and contextual covariates, compared with the low neighborhood disorder group, the HRs for recurrence in AMI patients living in middle and high neighborhood disorder groups were 1.03 (95 % CI: 0.98 to 1.07) and 1.08 (95 % CI: 1.03 to 1.14), respectively. A stronger association was noted for fatal recurrent AMI, with estimates of 1.06 (95 % CI: 0.96 to 1.16) and 1.21 (95 % CI: 1.10 to 1.34), respectively. Tests for linear trends revealed statistically significant increases in the risk of recurrent AMI (*P*_trend_ = 0.003) and fatal recurrent AMI (*P*_trend_ < 0.001) with increasing neighborhood disorder tertiles in the final models. Continuous analyses also indicated an association between neighborhood disorder and AMI recurrence, with a 3 % and 5 % higher risk of recurrent AMI (95 % CI: 1.02 to 1.04) and fatal recurrent AMI (95 % CI: 1.02 to 1.07), respectively, based on a per unit increase in neighborhood disorder (Table 2).

### 3.4 Subgroup analysis

The significant effect of neighborhood disorder was only observed in men (HR 1.04, 95 % CI: 1.02 to 1.06) for recurrent AMI‥ No significant effect of modification was identified among gender and other separate subgroups (Fig. S2).

### 3.5 Sensitivity analyses

Several sensitivity analyses were performed. First, when additionally adjusting income and driving distance to the nearest PCI-capable hospitals, patients living in high neighborhood disorder retained a significantly higher risk of recurrent AMI, especially fatal recurrent AMI, than those living in low neighborhood disorder (Table S2). Second, the association analysis was restricted to 39,023 incident AMI patients to check our main findings. Although the associations were attenuated, compared with the low neighborhood disorder, patients living in high neighborhood disorder had a significant 1.16-fold hazard of fatal recurrent AMI (Table S3). In addition, AMI patients living in high neighborhood disorder had a higher risk of recurrent AMI, fatal AMI, and nonfatal AMI at 5 years, with the association strongest for fatal AMI (Fig. S3).

## 4 Discussion

To the best of our knowledge, this is one of the earliest efforts to examine the association between exposure to neighborhood disorder and long-term recurrence risk among discharged AMI patients worldwide. In this city-wide patient cohort, we found that patients living in central urban areas characterized by serious neighborhood disorder appeared to experience a higher risk of AMI recurrence, especially fatal recurrent AMI, after discharge for AMI. Moreover, the harmful effects were mainly observed among females.

Our findings among urban Beijing AMI patients are consistent with a previous longitudinal study that demonstrated that each standard deviation increase in neighborhood disorder was associated with a 20 % increased risk of cardiovascular disease after adjusting for covariates among African American women (Barber et al., 2016). A recent metaanalysis identified three pathways from neighborhood disorder to adverse health outcomes, namely, provoking health-endangering behaviors, inspiring feelings of fear, and decreasing outdoor activities time caused by social and physical retreat (O’Brien et al., 2019a). To a certain extent, the pathways in the meta-analysis can explain why AMI patients living in areas with high levels of neighborhood disorder were more vulnerable to recurrent AMI. Rather than a single pathway, we believed that combined mechanisms existed. AMI patients living in areas with high levels of neighborhood disorder suffer from more stress (Plascak et al., 2021) and anxiety (Tang et al., 2017) and are more likely to exhibit behaviors of physical inactivity (Douglas et al., 2018), excessive alcohol and tobacco use (Hill & Angel, 2005), and more screen use (Carson & Janssen, 2012), which are associated with obesity (Cunningham-Myrie et al., 2015; Lovasi et al., 2012), decreased physical function, and more chronic health conditions (O’Brien et al., 2019a; O’Brien et al., 2019b; Ross & Mirowsky, 2001). In addition, in a more direct way, poor neighborhood built environment conditions also trigger the body’s natural mechanism of ‘fight or flight’, resulting in the release of glucose into the bloodstream caused by increased heart rate and blood pressure (Barber et al., 2016).

Another notable finding was that the effect of neighborhood disorder on recurrent AMI mainly appeared to be among females, which is similar to previous studies (Barber et al., 2016) that reported that the association between neighborhood disorder and the risk of CVD was more pronounced in women. In terms of behavioral risk factors for CVD, sex differences were also found in two cross-sectional studies that examined the association between neighborhood disorder and decreased physical activities (Miles, 2008) and excessive drinking (Kuipers et al., 2012). One hypothesis that may explain the harmful effects of neighborhood disorder among women was that women were affected more severely by deteriorated environmental conditions (Snedker, 2015), but they endured their vulnerability surreptitiously and hardly showed any remonstration (Rahman, 2013).

Previous studies assessing the relationship between the built environment and health outcomes usually focused on examining the exposure level towards specific facilities and places. In contrast, quality is a broader dimension of the built environment, existing along with every physical feature of the built environment. However, it is difficult to define a specific area as a low-quality built environment area (Miranda et al., 2012); thus, we measured the built environment quality based on the presence of physical features related to the visual aesthetics of the built environment using street view imagery. The features were divided into five domains, each of which was relatively independent and highly identifiable, providing extensive implications for designing the interventions. Features in architecture and retail categories captured the negative features that contributed to the architectural inaesthetic, describing an unfinished and informal condition of buildings. Most features in greening, road, and infrastructure categories described a destruction/declined condition of corresponding elements and facilities. The findings in our research suggested that, in addition to the pursuit of the proper density of and distance to health-promoting facilities, decision makers and urban planners should also focus on developing policies and regulations to remove neighborhood disorder features, eliminating inequalities in the quality of the built environment among different urban areas. Meanwhile, our study expanded the knowledge of the impact of neighborhood disorder on physical health. In particular, it allowed us to be aware of the harmful effect and potential link from neighborhood disorder to recurrent AMI and the harmful effect of neighborhood disorder on women, which was a strong support to the inconsistent evidence for neighborhood disorder’s impact on physical health (O’Brien et al., 2019a).

This study had several important strengths. First, the largest distinction from previous studies was that our neighborhood disorder focused on a range of visual-related features of the neighborhood environment, using common physical factors to reflect the quality of the built environment in the urban context. Compared with the study of Plascak et al. (2021), we have 10 more features in the neighborhood disorder measurement list. Our proposed neighborhood disorder definition could be beneficial for both future academic research on urban renovation and practical work that involved spatial management. Furthermore, using data from a city-level registry system, the study covered almost all AMI cases within the central Beijing area, which minimized selection biases. Additionally, the large sample size and longterm follow-up allowed us to comprehensively examine the adverse effects of neighborhood disorder on the long-term prognosis of AMI patients.

Several limitations should be considered in the interpretation of this study. First, we were not able to account or adjust for unidentified confounders, such as medication adherence and personal health behaviors, as we were limited by the availability of data resources. We acknowledge that there remains potential for residual confounding, although we have adjusted for various factors, including built environment risk factors (Malambo et al., 2016; Stevenson et al., 2016), PM_2.5_ exposure level (Lu et al., 2015), socioeconomic status (Ohm et al., 2021), and comorbidities, which have been reported to be associated with cardiovascular health in earlier studies. Second, due to the lack of images, this study used street view image data from 2015 to represent the mean exposure level of neighborhood disorder. Thirdly, our study focused solely on the built environment along the streets within the 500 m buffers, thereby overlooking the potential impact of human mobility on exposure measurement. To address this limitation in future research, we propose the use of wearable cameras to measure individual exposure to neighborhood physical disorder through a mobility-based approach (Li et al., 2022). These cameras can capture photos at short time intervals (e.g., every 10 s) and provide clear and accurate measurements of exposure during the daily activities.

## 5 Conclusion

This large-scale cohort study indicated that living in central urban areas characterized by serious neighborhood disorder is associated with a higher risk of recurrence in discharged AMI patients, and women were more prone to the harmful effects of neighborhood disorder. The findings from this study substantially expand current knowledge on the importance of neighborhood built environment quality on health outcomes, which can guide urban planners and policy makers to support the creation of high-quality urban neighborhood environments and healthy cities.

## Supplementary Material

Supplementary Materials

## Figures and Tables

**Table 1:** Baseline characteristics of index patients discharged with acute myocardial infarction in Beijing between 2007 and 2017.

Characteristics^a^	Total (n = 66,238)	Neighborhood disorder tertiles			*P*
		Low	Middle	High	
Age, years	66.0 (13.2)	66.5 (13.2)	66.5 (13.2)	65.2 (13.2)	<0.001
Age group, n (%)					<0.001
≤65 years	30,064 (45.4)	9574 (43.4)	9702 (43.9)	10,788 (48.9)	
>65 years	36,174 (54.6)	12,497 (56.6)	12,381 (56.1)	11,296 (51.2)	
Women, n (%)	19,273 (29.1)	6202 (28.1)	6531 (29.6)	6540 (29.6)	<0.001
Nonmarried, n (%)	4953 (7.5)	1664 (7.5)	1642 (7.4)	1647 (7.5)	0.909
Type of AMI, n (%)					
STEMI	37,339 (56.4)	12,340 (55.9)	12,374 (56.0)	12,625 (57.2)	0.002
NSTEMI	28,884 (43.6)	9730 (44.1)	9705 (44.0)	9449 (42.8)	
Unspecified	15 (0.0)	1 (0.0)	4 (0.0)	10 (0.1)	
History of CHD, n (%)	9167 (13.8)	3004 (13.6)	3111 (14.1)	3052 (13.8)	0.347
Comorbidities, n (%)					
Dyslipidemia	32,939 (49.7)	10,420 (47.2)	11,264 (51.0)	11,255 (51.0)	<0.001
Diabetes mellitus	24,417 (36.9)	7920 (35.9)	8213 (37.2)	8284 (37.5)	0.001
Hypertension	42,532 (64.2)	14,058 (63.7)	14,327 (64.9)	14,147 (64.1)	0.029
Residential location, n (%)					<0.001
South	32,170 (48.6)	4631 (21.0)	10,705 (48.5)	16,834 (76.3)	
North	34,015 (51.4)	17,411 (79.0)	11,366 (51.5)	5238 (23.7)	
Distance to the nearest park, m	729.1 (605.4)	598.5 (521.7)	721.3 (550.1)	898.2 (678.6)	<0.001
Distance to the main road, m	276.2 (363.4)	230.6 (309.8)	270.7 (367.3)	337.3 (395.1)	<0.001
PM_2.5_ exposure level, μg/m^3^	88.6 (1.5)	87.9 (1.3)	88.5 (1.4)	89.4 (1.4)	<0.001

AMI = acute myocardial infarction. STEMI=ST-segment elevation myocardial infarction. NSTEMI = non-ST-segment elevation myocardial infarction. CHD = coronary heart disease. PM_2_._5_ = particulate matter <2.5 μm in aerodynamic diameter.

a Values are means (standard deviations) for age and PM_2.5_, medians (interquartile ranges) for distance to the nearest park and distance to the main road, and counts (percentages) for other characteristics.

**Table 2:** Association between neighborhood disorder and recurrence risk after discharge for acute myocardial infarction patients.

Variable	Number of events	Hazard ratios (95%CI)		
		Model 1	Model 2	Model 3
Recurrent AMI event				
Low neighborhood disorder	3766	Reference	Reference	Reference
Middle neighborhood disorder	3963	1.06 (1.01-1.11)	1.06 (1.01-1.11)	1.03 (0.98-1.07)
High neighborhood disorder	4143	1.12 (1.07–1.17)	1.15 (1.10–1.20)	1.08 (1.03–1.14)
*p* for trend^a^		<0.001	<0.001	0.003
Continuous (per unit)		1.04 (1.03–1.05)	1.03 (1.03–1.05)	1.03 (1.02–1.04)
Fatal recurrent AMI event				
Low neighborhood disorder	969	Reference	Reference	Reference
Middle neighborhood disorder	1038	1.08 (0.99–1.18)	1.07 (0.98–1.17)	1.06 (0.96–1.16)
High neighborhood disorder	1110	1.16 (1.06–1.26)	1.25 (1.15–1.36)	1.21 (1.10–1.34)
*p* for trend^a^		0.001	<0.001	<0.001
Continuous (per unit)		1.03 (1.01–1.05)	1.06 (1.04–1.08)	1.05 (1.02–1.07)
Nonfatal recurrent AMI event				
Low neighborhood disorder	2797	Reference	Reference	Reference
Middle neighborhood disorder	2926	1.05 (1.00–1.11)	1.05 (1.00–1.11)	1.01 (0.96–1.07)
High neighborhood disorder	3034	1.10 (1.04–1.16)	1.11 (1.05–1.17)	1.03 (0.97–1.09)
*p* for trend†		<0.001	<0.001	0.318
Continuous (per unit)		1.03 (1.02–1.04)	1.03 (1.02–1.04)	1.02 (1.00–1.03)

Model 1 was not adjusted.

Model 2 was adjusted for age, sex.

Model 3 was adjusted for age, sex, marital status, type of AMI, history of coronary heart disease, comorbidities (dyslipidemia, diabetes mellitus, and hypertension), distance to the nearest park, distance to the main road, and PM_2.5_ exposure level.

AMI = acute myocardial infarction; CI = confidence interval.

a Based on a linear test for trends using the ordinal rank for each tertile.

## Data Availability

The authors do not have permission to share data.
