# Supplementary material for: Using street view imagery to examine the association between urban neighborhood disorder and the long-term recurrence risk of patients discharged with acute myocardial infarction in central Beijing, China
Source: Cities. Author manuscript; Available in PMC 2023 May 28. (PMC7614582; doi:10.1016/j.cities.2023.104366)
Supplement: Supplementary Materials [file EMS176143-supplement-Supplementary_Materials.docx]

# APPENDIX A Supplementary data

Supplementary table 1& figure 1 - The list of factors and virtual audit platform for neighborhood disorder

Supplementary table 2-3 & figure 2-3 Supplementary analysis

Table S1. The list of factors for neighborhood disorder.

| **Categories** | Factors | Samples |
| --- | --- | --- |
| 1. Architecture | 1.1 Abandoned Building | 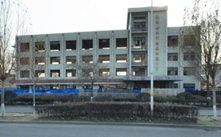 |
|  | 1.2 Half-demolished Building | 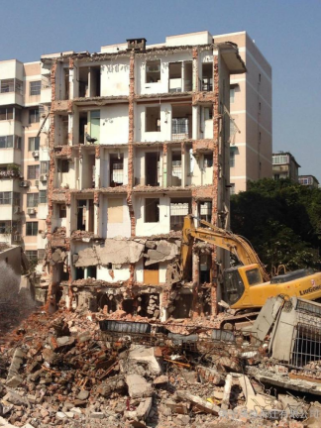 |
|  | 1.3 Broken Structure | 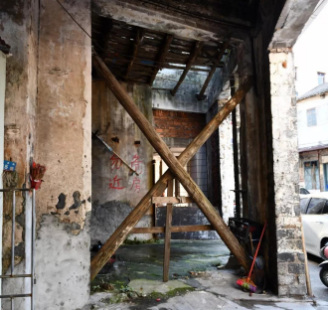 |
|  | 1.4 Unkempt Facade | 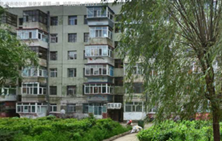 |
|  | 1.5 Graffiti/Advertisement | 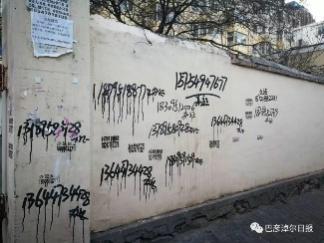 |
|  | 1.6 Illegal/Temporary Structure | 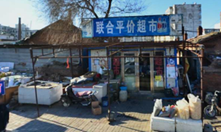 |
| 2. Retail | 2.1 Poor Signboard | 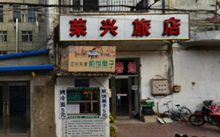 |
|  | 2.2 Poor Facade | 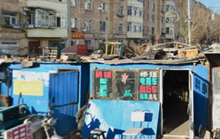 |
|  | 2.3 Roadside Stall | 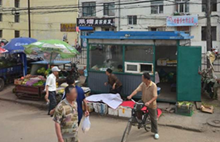 |
|  | 2.4 Vacant Store | 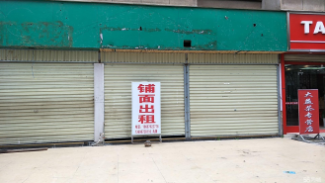 |
| 3. Greening | 3.1 Overgrown Plant | 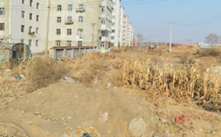 |
|  | 3.2 Trash, Litter, and Junk | 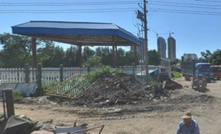 |
|  | 3.3 Abandoned Vehicle | 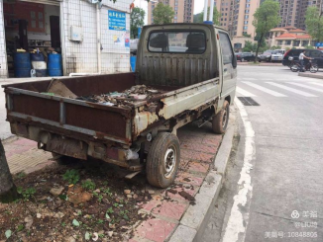 |
|  | 3.4 Construction Remnant | 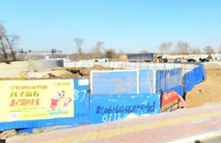 |
| 4. Road | 4.1 Unpaved Road | 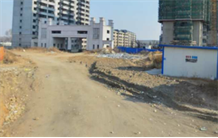 |
|  | 4.2 Broken Road | 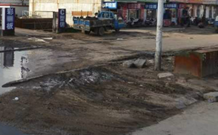 |
|  | 4.3 Trash Occupied Road | 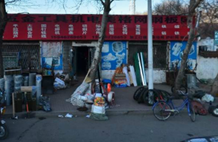 |
| 5. Infrastructure | 5.1 Broken Infrastructure | 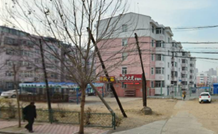 |
|  | 5.2 Rundown Public Interface | 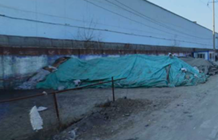 |

Table S2. Association between neighborhood disorder and recurrent risk after discharge for acute myocardial infarction patients, additionally adjusted for income and driving distance to PCI-capable hospital.

| Characteristics | Hazard Ratios (95%CI) | | |
| --- | --- | --- | --- |
|  | Model 3 +Income | Model 3  +Driving distance to PCI-capable hospital | Model 3  +Income +Driving distance to PCI-capable hospital |
| Recurrent AMI |  |  |  |
| Low neighborhood disorder | Reference | Reference | Reference |
| Middle neighborhood disorder | 1.03 (0.98-1.08) | 1.07 (1.00-1.13) | 1.07 (1.00-1.13) |
| High neighborhood disorder | 1.08 (1.02-1.14) | 1.12 (1.04-1.20) | 1.11 (1.04-1.19) |
| Fatal recurrent AMI |  |  |  |
| Low neighborhood disorder | Reference | Reference | Reference |
| Middle neighborhood disorder | 1.04 (0.94-1.15) | 1.11 (0.98-1.25) | 1.09 (0.97-1.24) |
| High neighborhood disorder | 1.19 (1.07-1.33) | 1.29 (1.14-1.48) | 1.30 (1.14-1.48) |
| Nonfatal recurrent AMI |  |  |  |
| Low neighborhood disorder | Reference | Reference | Reference |
| Middle neighborhood disorder | 1.02 (0.96-1.08) | 1.05 (0.97-1.12) | 1.05 (0.98-1.13) |
| High neighborhood disorder | 1.03 (0.97-1.10) | 1.05 (0.97-1.13) | 1.04 (0.96-1.13) |

AMI=acute myocardial infarction; CI=confidence interval; PCI=percutaneous coronary intervention.

Model 3 was adjusted for age, sex, marital status, type of AMI, history of coronary heart disease, comorbidities (dyslipidemia, diabetes mellitus, and hypertension), distance to the nearest park, distance to the main road, and PM_2.5_ exposure level.

Table S3. Association between neighborhood disorder and recurrent risk for incident acute myocardial infarction patients.

| Variable | Number of Events | Hazard Ratios (95%CI) | | |
| --- | --- | --- | --- | --- |
|  |  | Model 1 | Model 2 | Model 3 |
| Recurrent AMI event |  |  |  |  |
| Low neighborhood disorder | 1,962 | Reference | Reference | Reference |
| Middle neighborhood disorder | 1,938 | 0.99 (0.93-1.06) | 1.00 (0.94-1.06) | 0.96 (0.90-1.02) |
| High neighborhood disorder | 2,078 | 1.06 (1.00-1.13) | 1.12 (1.05-1.19) | 1.03 (0.96-1.10) |
| Fatal recurrent AMI event |  |  |  |  |
| Low neighborhood disorder | 499 | Reference | Reference | Reference |
| Middle neighborhood disorder | 492 | 0.99 (0.87-1.12) | 1.00 (0.89-1.14) | 0.98 (0.86-1.12) |
| High neighborhood disorder | 543 | 1.09 (0.97-1.24) | 1.22 (1.08-1.38) | 1.16 (1.01-1.33) |
| Nonfatal recurrent AMI event | |  |  |  |
| Low neighborhood disorder | 1,463 | Reference | Reference | Reference |
| Middle neighborhood disorder | 1,446 | 0.99 (0.92-1.07) | 1.00 (0.93-1.07) | 0.95 (0.89-1.03) |
| High neighborhood disorder | 1,536 | 1.05 (0.98-1.13) | 1.08 (1.00-1.16) | 0.98 (0.91-1.07) |

Model 1 was not adjusted.
Model 2 was adjusted for age, sex.
Model 3 was adjusted for age, sex, marital status, type of AMI, history of coronary heart disease, comorbidities (dyslipidemia, diabetes mellitus, and hypertension), distance to the nearest park, distance to the main road, and PM_2.5_ exposure level.
AMI=acute myocardial infarction; CI=confidence interval.


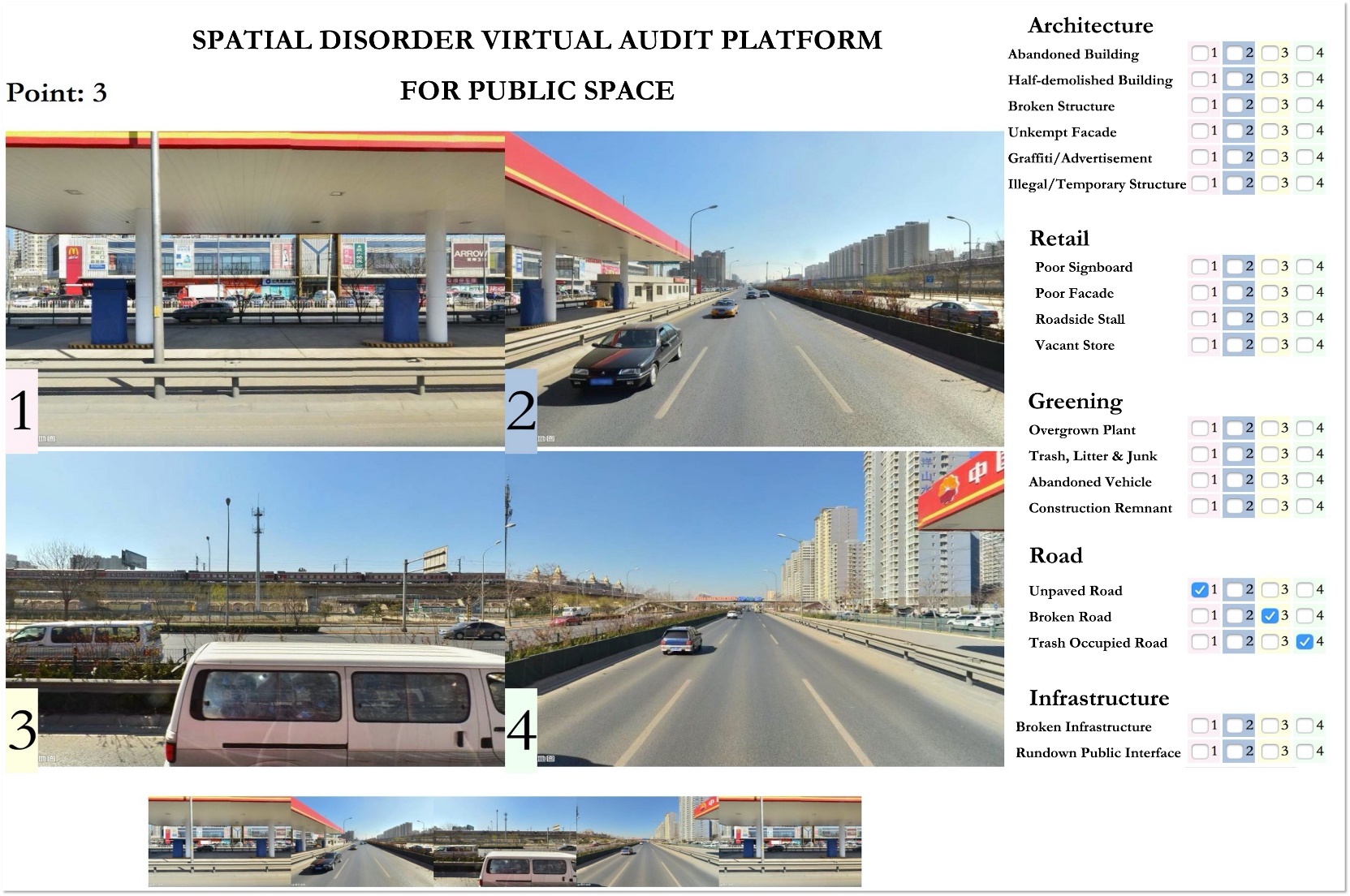
Figure S1. Virtual audit platform for neighborhood disorder.

Figure S2. Adjusted hazard ratios for the risk of recurrent AMI and fatal recurrent AMI per unit neighborhood disorder, according to baseline characteristics.

CI=confidence interval, AMI=acute myocardial infarction, STEMI=ST-segment elevation myocardial infarction, NSTEMI=non-ST-segment elevation myocardial infarction, CHD=coronary heart disease, PM_2.5_=particulate matter < 2.5 μm in aerodynamic diameter. *P* values were calculated using a two-sample z-test to assess subgroup-specific effects.

Figure S3. Association between neighborhood disorder and recurrence risk at 5 years after discharge for acute myocardial infarction patients.

* The hazard ratio was adjusted for age, sex, marital status, type of AMI, history of coronary heart disease, comorbidities (dyslipidemia, diabetes mellitus, and hypertension), distance to the nearest park, distance to the main road, and PM_2.5_ exposure level.

† Based on a linear test for trends using the ordinal rank for each tertile.
AMI=acute myocardial infarction; CI=confidence interval.
